# Ophthalmic Diseases and Therapeutics

**Published:** 1893-01

**Authors:** 


OPHTHALMIC DISEASES AND THERAPEUTICS. By A. B. Norton, M. D., with fifty-three illustrations and twelve chromolithographic figures. Philadelphia: Bœricke & Tafel, 1892. Price, $3.50 net.

This is a neat volume of five hundred and fifty-five pages which the author has dedicated to the late George S. Norton, M. D. It contains an admirable and striking likeness of him which will be highly prized by the profession.

The work is divided into two parts; part I entitled Ophthalmic Diseases, and part II Ophthalmic Therapeutics. In part I the author has given us a very concise and clear description of eye diseases, mention being made after the description of each disease of the remedies which, in the author's opinion, are most frequently indicated, together with some indications for their use. In a few words and in a very condensed form he has written an interesting description of these diseases without in the least slighting the subject. Part

II is devoted to Ophthalmic Therapeutics, being the ocular pathogenesis of each remedy in alphabetical order.

In appearance and in arrangement it resembles the *Ophthalmic Therapeutics* by the late George S. Norton, M. D., but although it is, as the author states, designed to be a continuation of that work, still it has been so much enlarged and improved as to make it a work vastly superior to it in every respect. But there are two things omitted from this work which, had it contained it, would have made it of much more value to the practitioner. There is no repertory and no mention is made of concomitant symptoms.

